# The Air Permeability and the Porosity of Polymer Materials Based on 3D-Printed Hybrid Non-Woven Needle-Punched Fabrics

**DOI:** 10.3390/polym16101424

**Published:** 2024-05-17

**Authors:** Victor Nazarov, Alexander Dedov, Fedor Doronin, Mikhail Savel’ev, Andrey Evdokimov, Georgy Rytikov

**Affiliations:** Faculty of Printing Industry, Moscow Polytechnic University, 107023 Moscow, Russia; 110505n@gmail.com (V.N.); dedovs55@rambler.ru (A.D.); vidogo@yandex.ru (M.S.); evdokag@gmail.com (A.E.); gr-yandex@yandex.ru (G.R.)

**Keywords:** additive manufacturing, material extrusion screen printing, non-woven polymer fabric, bicomponent fiber, modification, air permeability, porosity

## Abstract

The possibility of controlling the porosity and, as a result, the permeability of fibrous non-woven fabrics was studied. Modification of experimental samples was performed on equipment with adjustable heating and compression. It was found that the modification regimes affected the formation of the porous structure. We found that there was a relationship between the permeability coefficient and the porosity coefficient of the materials when the modification speed and temperature were varied. A model is proposed for predicting the permeability for modified material with a given porosity. As the result, a new hybrid composite material with reversible dynamic color characteristics that changed under the influence of ultraviolet and/or thermal exposure was produced. The developed technology consists of: manufacture of the non-woven needle-punched fabrics, surface structuring, material extrusion, additive manufacturing (FFF technology) and the stencil technique of ink-layer adding. In our investigation, we (a) obtained fibrous polymer materials with a porosity gradient in thickness, (b) determined the dependence of the material’s porosity coefficient on the speed and temperature of the modification and (c) developed a model for calculating the porosity coefficient of the materials with specified technological parameters.

## 1. Introduction

Non-woven needle-punched materials (used for gas and liquid filtration [1,2,3,4], heat [4,5,6,7,8] and sound insulation [7,8,9,10,11], and road and hydraulic engineering construction [11,12,13,14], etc.), must have high permeability and sufficient mechanical properties for practical uses that depend on the fabric’s porosity [15]. Gas and liquid permeability increases with higher porosity [15,16,17], but the mechanical properties tend to deteriorate [18,19,20].

We have previously developed the prescription and technological solutions [21,22,23,24] for the obtaining of materials with the adjustability [25,26,27,28] for practical use: permeability [23] and sufficient tensile and compression resistance [24]. Non-woven needle-punched fabrics (made of polyethylene terephthalate [29,30] and bicomponent fiber [31] mixtures) were used for the modification. The modified materials with gradient pores (their dimensions varied in thickness [21]) were obtained as the result of thermal and mechanical treatment.

The optimal combination of permeability and mechanical properties of the modified materials was achieved when using non-woven fabrics made of a 70:30 weight% ratio of polyethylene terephthalate and bicomponent fibers. However, the technology of these important modified polymers with specific structural production has not previously been investigated.

Another urgent scientific and technical task is the creation of hybrid materials through a combination of additive prototyping technologies and manufacturing techniques for the production of the structured non-woven fabric-based goods (biostable membranes, air filters, sound and heat insulation, radio-absorbing materials, geotextiles, etc.).

A new extrusion additive prototyping technique is proposed in [32]. It allows for the creation of hierarchical systems (consisting of porous layers of nano-/microfibers) using a modernized fused filament fabrication (FFF) 3D-printer printhead in (a) filament-welding or (b) fiber-generation modes. The fiber generation was carried out with hot air under pressure blowing through the polymer melt and stretching it into the thin fibers. The use of PolyJet additive prototyping technology to produce combined materials with 3D-printed structures on a textile fabric base was first comprehensively considered in [33]. The application of 3D prototyping for the production of functional non-woven materials with phase transition (PCNF) and adjustable pores was demonstrated in [34]. The use of 3D-printed mesh structures for biomedical applications and wearable devices was shown in [35]. The possibility of 3D-printed structures forming with stereolithography (SLA) technology on the textile fabrics was demonstrated in [36] for the first time. The effect of the surface structure on the adhesion of the UV-cured pitch layers has been established. A set of techniques for gas-phase surface structuring of various polymer materials (providing the ability to control their functional properties) is described in [37,38,39].

The aim of our work was to study the effect of the surface density of non-woven fabrics on the formation of a porous structure and the air permeability of the modified hybrid materials which are manufactured using FFF-3D-printing technology.

## 2. Materials and Methods

The objects of the study were the non-woven needle-punched webs formed from a mixture of polyethyleneterephthalate (JSC Mogilevkhimvolokno, Mogilev, Republic of Belarus) and bicomponent fibers (Samsung, Suwon, Republic of Korea) in a ratio of 70:30 wt.%. Grade D polyethylene terephthalate was used for fiber production. The specific viscosity of the polymer in dichloroacetic acid was 827–880 units, the titanium mass fraction was 0.28–0.32% and the melting point was −261 °C. The number of COON groups was not more than 30 mmol/kg. The mass fraction of diethylene glycol did not exceed 0.8%. The commercial name of the bicomponent fiber is HUVIS.

The linear densities of the fibers were 0.33 tex (diameter 20–25 microns) and 0.44 tex (diameter 30–33 microns). Bicomponent fiber has a core–shell structure [31]: the core consists of a high molecular weight polyethyleneterephthalate with a melting point of 250–270 °C and the shell is made of low molecular weight polyethyleneterephthalate with a melting point near 110–120 °C.

**Figure 1:**
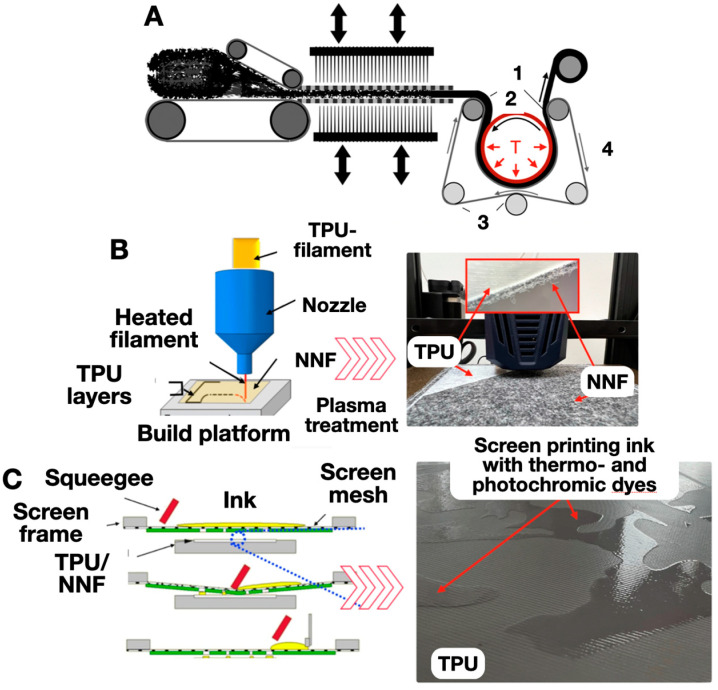
The technology and the stages (**A**–**C**) of the hybrid experimental samples (based on the non-woven needle-punched fabric (NNF) and material extrusion additive TPU filament) manufacturing. 1—Modified material; 2—heated shaft; 3—guide shafts; 4—conveyor belt (a thin arrow shows the direction of movement of the conveyor belt and a thickened arrow shows the movement of the fabric); T—heat.

The main stages of the hybrid experimental samples (based on the non-woven needle-punched fabrics (NNFs) and thermoplastic polyurethane FLEX U3Print TPU 60D (U3Print, Klin, Russia)) manufacturing were (Figure 1): (A) Formation of a non-woven fabric from PET fibers and needle punching and deformation–thermal treatment of the NNF.

The fibrous fabric was obtained by mechanical molding at the industrial installation of DiloSpinnbau (Eberbach, Germany). Its hardening was performed using a needle punching unit (Di loDi-Loom LADIES 50 (Eberbach, Germany)). The density of the main puncture was 180 cm^−2^. The thermomechanical treatment of the cartridge was carried out using a calendared thermal press MA-76 from Monti Antonio (Thiene, Italy).

(B) Application of TPU coatings to the NNF surface by 3D extrusion prototyping (3D printer Anycubic Kobra Go. (Shenzhen, China), nozzle temperature 210 °C, 3D-printing speed—30 mm/min). (C) Screen printing (screen-printing machine Carousel 4S Argon HT (Via Casottina, Italy)) containing energychromes (thermo- and photochromes) of the ink functional layers on the surface of the initial and the plasma–chemically treated (atmospheric plasma system Diener Plasma APC 500 (DienerelectronicgGmbH&Co. KG, Ebhausen, Germany)) experimental samples. Atmospheric air (79 volume% nitrogen and 21 vol. % oxygen) was used as a plasma-forming gas. The power of the plasma generator was 500 W (frequency 40 kHz).

Energychromic (thermo- and photochromic) inks were used: screen-printing ink (Nylotex NX SericolFujifilm (Tokyo, Japan) with 10 weight% of thermochromic (black and green) ink), or photochromic (magenta and yellow) pigments (produced by Hali Industrial Co., Ltd. (Changzhou, China)).

The formation of the non-woven webs was carried out mechanically using a specialized device produced by the Spinbau Carding Unit (Bremen, Germany). Reinforcement (with the needle-punching technique) was then performed on a Dilo unit (Babenhausen, Germany), with varying piercing densities. The thermomechanical modification of the non-woven webs (1) was carried out with the help of the original experimental equipment (Figure 1). The shaft (2) had a protective fluorinated rubber that limited the adhesion of the synthetic fibers to its surface. The conveyor belt (4) was made of aramid fibers by piercing a looped web without a seam with an operating temperature of up to 250 °C. The pressure on the web in the gap between the heated shaft (2) and the conveyor belt (4) was regulated by the position of the guiding shafts (3). Their movements limited the transverse shift of the conveyor belt (4). The tension on the shafts (3) was created by the pneumatic cylinders. The deformation and thermal effects were exerted directly in the gap between the heated shaft (2) and the conveyor belt (4). In different experiments, the temperature of the shaft was 110, 130, 150, 160, 170, 185 or 200 °C, and the speed of the web was 1.2, 1.5, 2.5, 3.0, 5.0, 6.0, 9.0, 10 or 12 m/min. The temperature change in the transported material during processing was determined by the duration of its direct contact with the heated shaft. When leaving the contact zone, blowing of intensive cold air led to fixation of the sample structure due to its effective cooling. The compressive forces in the gap between the shaft and the belt helped to reduce the degree of the longitudinal stretching for both the original and the modified non-woven webs.

The coefficient *δ* was used to quantify the porosity of the experimental samples:

(1) $$\delta =1-\frac{p}{p_{f}}$$

where *p* (kg/m^3^) is the average density of the non-woven web and *p*_f_ (kg/m^3^) is the average density of the fiber material (for polyethylene terephthalate and bicomponent fibers it was ~1370 kg/m^3^).

A coefficient *K* (m^2^) was used to quantify the air permeability of the initial and modified materials:

(2) $$K=\frac{w_{B}\cdot \eta \cdot d}{∆P}$$

This coefficient was obtained from the equation $w_{B}=\left(K\cdot ∆P\right)/\left(\eta \cdot d\right)$ [14,15,16], in which *w_B_* (m/s) is the air filtration rate; ∆*P* (Pa) is the air pressure difference (the tests were carried out at ∆P~49 Pa); *d* (m) is the average thickness of the initial and the modified webs and *η* (Pa × s) is the air viscosity (it was assumed to be equal to 1.8 × 10^5^ Pa × s at a temperature of 20 ± 2 °C). The relative error of the air permeability coefficient for the initial and modified materials was no more than 8%.

We then studied the possibility of thermochromic dyes causing dynamic and reversible changes of the TPU/NNF-based textile products’ color. Color coordinate measurement of the inked surface elements of the experimental samples was carried out in the CIE L*a*b* color space using an X-Rite eXact spectrophotometer. The quantitative assessment of the color difference ∆*E* was calculated in accordance with formula:

(3) $$∆E^{*}=\sqrt{{\left(∆L^{*}\right)}^{2}+{\left(∆a^{*}\right)}^{2}+{\left(∆b^{*}\right)}^{2}}$$

(4) $$∆L^{*}=∆L_{T}^{*}-∆L_{S}^{*};\;∆a^{*}=∆a_{T}^{*}-∆a_{S}^{*};\;∆b^{*}=∆b_{T}^{*}-∆b_{S}^{*}$$

The reflection spectra were obtained under the following constant conditions: illumination—D65, viewing angle (observer)—2°, density measurement according to the standard (density)—DIN, calibration standard—white base abs, and filter—Pol. The recording time of the reflection spectrum was less than 1 s, and the resolution of the spectrophotometer was ~10 nm. The tensile strength of TPU/NNF was determined using a universal Zwick Roell BZ1.0 tensile testing machine. The RM-50 bursting machine (Mashplast, LLC, Moscow, Russia) was used to determine the tear strength σ (adhesion) of the ink layer to the surface of the TPU/NNF sample. A metal cylinder with a butt area of 1 cm^2^ was attached to the upper clamp of the RM-50 by means of a flexible rod and the strength values were recorded in the Stretch Test program.

## 3. Results and Discussion

**Figure 2:**
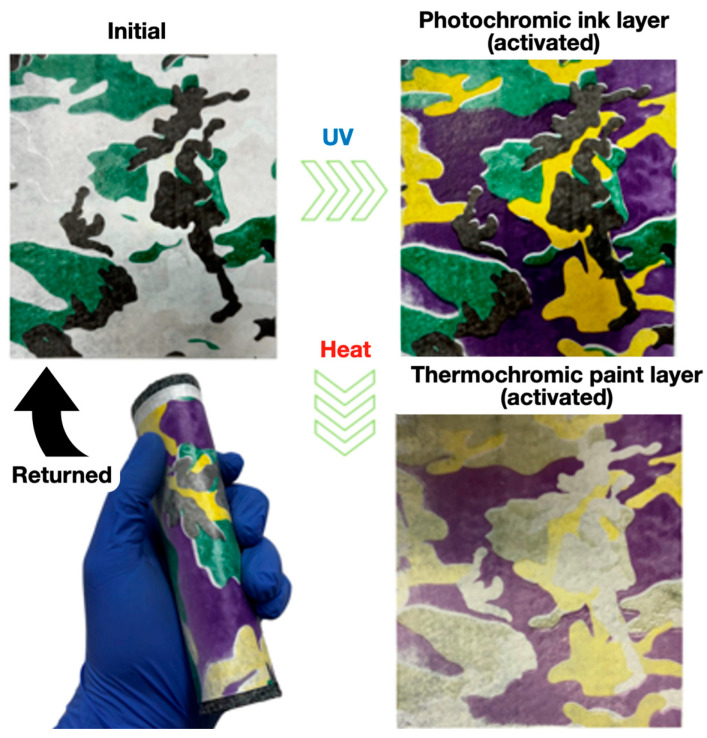
The reversible change in the color characteristics of an experimental TPU/NNF sample under the influence of the environmental factors (heating for 20 s + UV radiation). The TPU/NNF-sample returned to its initial state within ~2 min (Table 1).

The reversible dynamic changes in the color characteristics of the hybrid (TPU/NNF) experimental samples (made by layered deposition of TPU filament on the effective surface of non-woven needle-punched fabrics (NNFs)) inked with the energychromic (thermo- and photochromic) dyes was demonstrated. The results of the UV radiation (365 nm, 5 s) and thermal exposure (80 °C, 20 s) effect on the painted TPU/NNF samples’ surfaces are shown in Figure 2 and Table 1.

It was found (Table 2) that the experimental TPU/NNF samples withstood multiple twisting due to an 2.8-fold increase in interlayer adhesion (from 0.3 to 0.84 MPa) in the “filament-layer−printing ink” pair. They could also reversibly change their color characteristics depending on the influence of the environmental factors (heating and/or UV radiation) (Figure 2). We increased the strength characteristics of TPU/NNF by ~3.5 times compared with the initial values due to the optimization of the NNF-manufacturing technology by our rational rolling-speed choice. As a result, the adhesion reliability of the additive layers for the TPU filament to the NNF’s PET fibers was increased (Table 2).

The results of measurements and calculations (the average values of the porosity coefficient δ (relative units), thickness d (mm), surface F (kg/m^2^) and volumetric p (kg/m^3^) density obtained for three classes of the experimental samples) are presented in Table 3.

**Figure 3:**
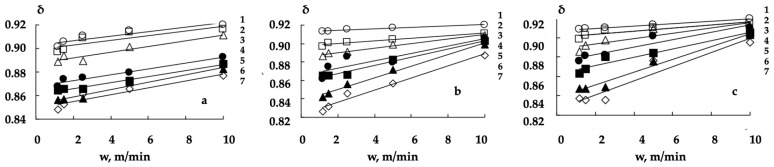
The porosity coefficient δ dependences on the non-woven webs (with surface densities of 0.15 (**a**), 0.25 (**b**) and 0.40 (**c**) kg/m^2^), processing speed w (m/min), at shaft temperatures 110 (1), 130 (2), 150 (3), 160 (4), 170 (5), 185 (6) and 200 (7) °C.

The porosity coefficient δ mean the values’ dependences on the speed w (m/min) of the non-woven webs’ (with different surface densities, F) thermomechanical processing (at 110, 130, 150, 160, 170, 185 and 200 °C) are shown in Figure 3.

An increase in the shaft temperature and a decrease in the processing speed led to the formation of thermomechanically modified non-woven webs with a reduced porosity coefficient. It can be seen (Figure 3a–c) that the character of the dependences of the porosity coefficient on the parameters of the processing mode depended on the non-woven webs’ surface density. The δ on w dependences’ approximating lines for the materials with a surface density of 0.15 kg/m^2^ are practically parallel (Figure 3a). This reflects a proportional decrease in the porosity coefficient with an increase in the shaft temperature.

The range of the porosity coefficient variation at different processing speeds also depended on the shaft temperature for the non-woven webs with a surface density of 0.25 and 0.40 kg/m^2^. However, the change in the processing speed from 1.2 to 10 m/min had little effect on the porosity coefficient at a shaft temperature below 160 °C. A decrease in the processing speed led to a decrease in the porosity coefficient at a shaft temperature above 160 °C. In general, the higher the shaft temperature the more the changes in thermomechanical processing speed affected the changes in the porosity coefficient (Figure 3b,c).

The observed relationship between the porosity coefficients’ values and the thermomechanical treatment modes’ parameters are determined by the induced changes in the structure of the materials under consideration. The shell of bicomponent fibers begins to melt at a certain temperature. When compressing a non-woven fabric in the gap between the heated shaft and the conveyor belt, the bicomponent fibers are fused with polyethylene terephthalate and with each other. The consequence of spatial fixation (as a result of cooling) of new (formed by fused fibers) volumetric structures of the modified non-woven web was a decrease in the porosity coefficient and irreversible compression deformation of the corresponding experimental samples (Figure 3a–c).

The δ on w dependences (Figure 3) are described with the linear equations at an acceptable level of quality (*R*^2^ > 0.7):

(5) δ=A×w+B

where, for a fixed shaft temperature, A (min/m) coefficient determines the effect of the processing speed on the porosity coefficient and B (related units) coefficient allows us to estimate the porosity value at an infinitesimal non-woven fabric processing speed (w→0).

**Figure 4:**
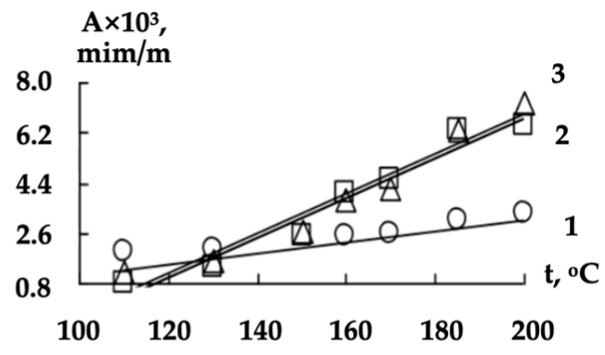
The dependences of A coefficient (3) on the shaft temperature when processing the non-woven webs with a surface density of 0.15 (1), 0.25 (2) and 0.40 (3) kg/m^2^.

**Figure 5:**
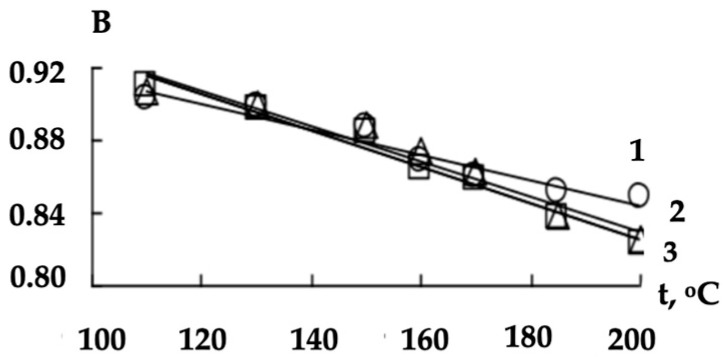
The dependences of B coefficient (4) on the shaft temperature when processing the non-woven webs with a surface density of 0.15 (1), 0.25 (2) and 0.40 (3) kg/m^2^.

We also investigated the character of the linear models’ (5) parameters (A- and B-dependences) on the shaft temperature (Figure 4 and Figure 5).

The revealed dependences of *A* and *B* coefficients on the shaft temperature *t* (°C) were also approximated by the linear functions:

(6) A=C×t+D

(7) B=L−R×t

The obtained values of the *C*, *D*, *L* and *R* coefficients are presented in Table 4.

We obtained Equation (8) as a result of substituting (6) and (7) in (5). This allowed us to predict the porosity coefficient values of the modified non-woven webs and to carry out a preliminary assessment of the potential effectiveness of previously unused thermomechanical treatment modes:

(8) $$\delta =\left(C\times t+D\right)\times w+\left(L-R\times t\right)$$

**Figure 6:**
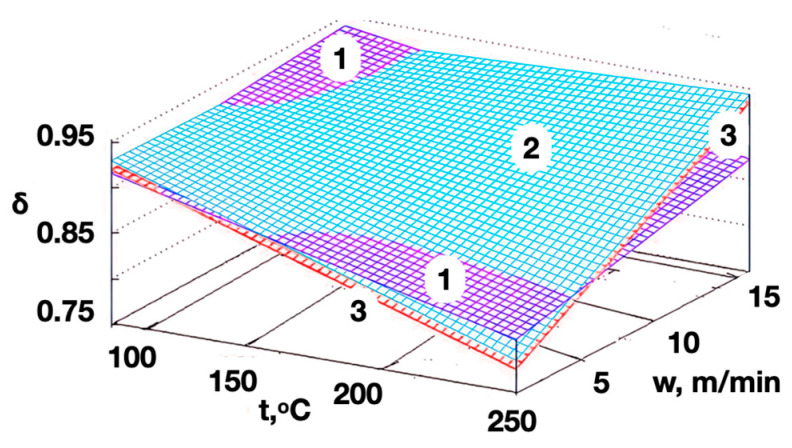
The dependence of the modified materials’ porosity coefficient on the shaft temperature and on the processing speed for the non-woven webs with surface densities of 0.15 (1), 0.25 (2) and 0.40 (3) kg/m^2^.

The result of the porosity coefficient values’ calculation using model (8), with a wide range of shaft temperature from 100 to 250 °C, and a material-processing speed from 1 to 16 m/min are visualized in Figure 6.

The non-woven webs with a surface density of 0.15 kg/m^2^ (processed at a combination of shaft temperatures above 150 °C and a web transportation speed of less than 5 m/min) had the maximum porosity. The porosity coefficient of the modified materials with a surface density of 0.15 kg/m^2^ (with increasing the processing speed) was lower than the coefficients of modified materials with a surface density of 0.25 or 0.40 kg/m^2^ (Figure 6, dependence 1). The porosity coefficients of the modified non-woven webs with surface density of 0.25 or 0.40 kg/m^2^ (treated under the same technological modes) had similar values (Figure 5, dependences 2 and 3, respectively). The porosity coefficients of the modified materials with different surface densities differed little at a shaft temperature below 150 °C and a change in the processing speed from 1 to 16 m/min.

The air permeability coefficient of thermomechanically modified non-woven webs is mainly determined by their porosity that (as was shown) depends on the surface density of the initial material and the speed and temperature of its processing.

**Figure 7:**
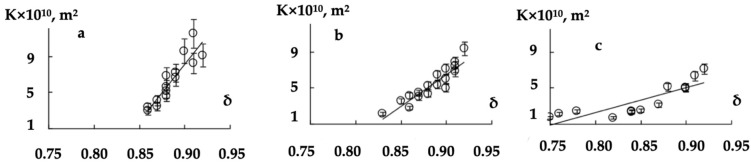
The permeability on the porosity dependences for the modified materials based on the non-woven webs with the surface densities of 0.15 (**a**), 0.25 (**b**) and 0.40 (**c**) kg/m^2^.

The air permeability dependences of the modified non-woven webs with different surface densities on their porosity coefficient are shown in Figure 7.

Each point corresponds to the average value of the permeability coefficient of a series of samples characterized by the same porosity. The relative error of the permeability coefficient mean value depended on the surface density of the initial web and its processing modes. For different surface densities, modified non-woven webs with a porous structure did not differ significantly from the initial one and the relative error increased when the porosity coefficient grew.

The use of the permeability coefficient mean value for the quantitative description of K on δ dependences (Figure 7) was possible in cases where the deviation (which was ~8%) of the dependent variable value from the calculated one was less than the relative error of the experiment (which was ~14%). The dependence of the permeability coefficient relative error on the porosity of thermomechanically modified materials is a consequence of heating, cooling, deformation and shear of fibers during the formation of the non-woven web porous structure.

Dependencies of *K* on *δ* for thermomechanically modified materials with different surface densities were also approximated by the linear functions:

(9) $$K\times 10^{10}=N\times \delta +K_{0},\;\delta >\delta_{cr}$$

where *δ* is the porosity coefficient, *K*_0_ (m^2^) is the permeability coefficient of the material at *δ*→0 case, and *N* (m^2^) is a quantitative characteristic of the porosity influence degree on the materials air permeability.

Extrapolation of the K on δ dependencies to the region of small porosity coefficient values suggests the presence of a minimum porosity coefficient value δ_cr_ at which the air permeability becomes equal to zero. If the porosity coefficient is less than δ_cr_ then the model loses its physical meaning since the permeability coefficient K should not take negative values. The values of the coefficients N, K_0_ and δ_cr_ obtained for the non-woven webs with different surface densities are presented in Table 5.

It follows from the experimental data (Table 5) that δ_cr_ decreases with an increase in the surface density F of the non-woven web. This indicates the influence of the porosity of the surface layer on the air transport in the bulk of modified samples.

**Figure 8:**
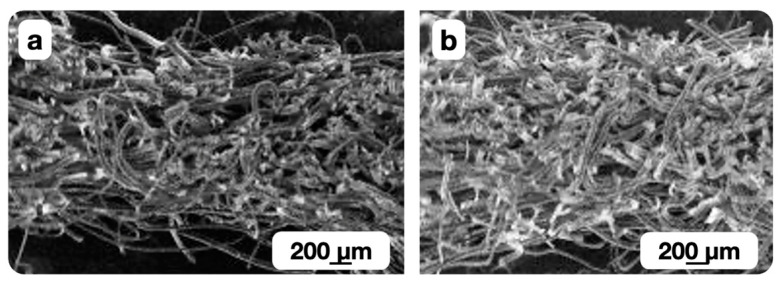
Micrograph of the modified materials with a surface density of 0.15 kg/m^2^ with a porosity coefficient of less than (**a**) and more than (**b**) 0.86 (0.82 and 0.88, respectively).

A significant amount of the heat is removed from the surface layer into the bulk during the thermomechanical processing of the non-woven webs with a higher surface density. The formation of pores with complex shapes (in which the fibers are arranged randomly) was seen most often in modified webs with a surface density of 0.15 kg/m^2^. This manifested in a larger relative error of the permeability coefficient mean value. The overheating of the surface layer bicomponent fibers led to fixation of the fused fibers in a deformable state, and to the effective pore formation (Figure 8b).

The insufficient heating of bicomponent fibers in the surface layer limited the possibility of fixing their shape in a deformable state as a result of cooling. So the fibers were restored to their original shape when the material left the zone of thermal and deformation actions. The spatial fixation of the non-woven web structure with the melt of the bicomponent fibers’ shell did not occur (Figure 8a) for the materials with a porosity coefficient of more than 0.86 obtained at a relatively low shaft temperature and high processing speed. The corresponding fibers shifted and filled the pore volume in the gap between the heated shaft and the conveyor belt.

The structures of the modified surface layers with a porosity coefficient of less than (a) and more than (b) 0.86 (0.82 and 0.88, respectively) are shown in Figure 8a,b, respectively.

**Figure 9:**
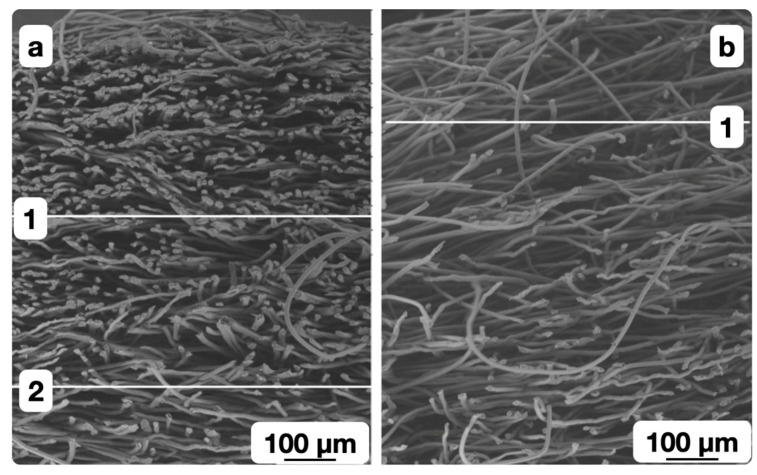
Micrographs of the modified materials with a surface density of 0.15 kg/m^2^ and a porosity coefficient of 0.72 (**a**) and 0.90 (**b**) (the modified layers are located above line 1; (**a**) shows a transition layer between lines 1 and 2).

The increase in density of the treated materials was a consequence of the compaction of the modified surface layer compaction. This conclusion is based on the fact that the volume of processed materials differed very little for the samples with a porosity coefficient of 0.72 (Figure 9a) and 0.90 (Figure 9b). The material with a porosity of 0.90 (Figure 9b) had a noticeable interface between the modified layer and the volume. A transition layer (between the modified layer and the volume) with the intermediate fiber packing density (less than one for the modified layer, but greater than one for the material’s volume) was observed for the material with a porosity of 0.72.

The regulation of fiber packing density in the modified layer is of practical importance for the filtration of different gases and liquids. Moreover, it becomes possible to produce filter materials with a high ability to retain the solid particles on the surface, which is necessary for the subsequent regeneration of the material.

## 4. Conclusions

A new hybrid composite material (TPU/NNF) was obtained as a result of the consistent application of optimized manufacturing technologies (composite non-woven needle-punched fabrication, surface structuring, 3D extrusion prototyping with TPU-filament and the stencil technique of applying an ink layer). The TPU/NNF samples withstood repeated twisting and were characterized by a complex of improved functional and operational properties: the modulus of elasticity and mechanical strength were ~3.5 times larger than the initial ones and the adhesion in the “filament layer–printing ink” pair increased by ~3 times (from ~0.30 to ~0.84 MPa). The resulting composite materials were able to reversibly change their color characteristics depending on the environmental factors (heating and/or UV-radiation) due to the use of thermo- and photochromic ingredients in the composition of printing inks. The developed hybrid composite materials are usable in textiles, for decorative purposes and as a “smart” material capable of adapting its color to that of the surrounding background at different light levels and temperatures. Such a material can be a visual indicator of changes in the temperature of the objects and/or the environment. It can be modified further (due to impregnating with various liquid compositions) to improve the mechanical and/or functional properties (due to the porosity and, as a result, the gas and liquid permeability).

The surface density determined the relationship of the non-woven webs’ heating and the bicomponent fibers’ shell melting temperature and the thickness of the layer in which the irreversible deformation occurred. It also results in a decrease in the modified material’s porosity and must be taken into account when obtaining non-woven materials with a given porous structure. The porosity coefficient decreased at a shaft temperature above 160 °C for the non-woven webs with a surface density of 0.25 and 0.40 kg/m^2^; the lower the processing speed, the more the porosity of the modified materials decreased. The character of air transport in the initial and the modified web was maintained until a certain value of the porosity coefficient was reached; this value decreased with an increase in the materials’ surface density.

## Figures and Tables

**Table 1:** The results of color characteristics measurements for the colorful layers on the TPU/NNF surface before and after exposure to UV radiation and/or heating.

Color Characteristics	L	a	b
TPU/NNF (initial surface)	89.28	−3.93	2.17
	∆L	∆a	∆b
Initial colorful green thermochromic layer	53.11	−49.67	19.04
Initial colorful black thermochromic layer	28.32	1.9	3.2
Initial colorful yellow thermochromic layer (before exposure to UV)	89.82	−4.37	5.05
Initial colorful purple photochromic layer (before UV exposure)	83.76	0.31	−1.47
Colorful yellow photochromic layer (colored after UV exposure)	75.62	11.31	75.73
Colorful purple photochromic layer (colored after UV exposure)	26.51	41.28	−25.68
Colorful green thermochromic layer (discolored)	81.94	−7.83	7.57
Colorful black thermochromic layer (discolored)	83.7	−3.54	4.25

**Table 2:** The results of mechanical and adhesive property tests for the NNF and TPU/NNF samples depending on the rolling speed of the non-woven needle-punched fabrics.

**Sample**	**Tensile Strength σ, MPa**					
	Rolling Speed, m/min					
	0	1.5	3	6	9	12
NNF	1.7	2.28	3.3	2.83	2.77	3.1
TPU/NNF	-	9.0	10.5	10.7	9.4	10.2
	Tear strength of printing ink from the surface of TPU/NNF, MPa					
Initial	0.30			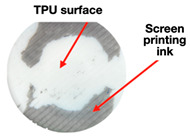 Adhesive destruction of the ink layer		
Plasmochemical treated for 60 s	0.84			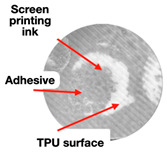 Adhesive−cohesive destruction of the ink layer		

**Table 3:** The average values of the porosity, thickness, surface and bulk density for three classes of experimental samples.

F, kg/m^2^	d × 10^3^, m	P, kg/m^3^	δ, Relative Units
0.15	1.5	100.0	0.93
0.25	2.2	113.6	0.92
0.40	3.23	116.1	0.92

F (kg/m^2^) is the surface density; d (m) is the thickness; p (kg/m^3^) is the bulk density; δ (relative units) is the porosity coefficient.

**Table 4:** Empirically established values of the coefficients of Equations (6) and (7) for the modified materials with different surface densities.

F, kg/m^2^	C × 10^5^, (°C)^−1^	D × 10^4^	L	R × 10^3^, (°C)^−1^
0.15	1.6	0.4	0.98	7.0
0.25	7.1	−75	1.03	1.0
0.40	7.2	−76	1.04	1.1

F is the surface density; C, D, L and R are the coefficients of Equations (6) and (7).

**Table 5:** The empirically established mean values of Equation (9) coefficients and the corresponding critical values of the porosity coefficient.

F, kg/m^2^	N, m^2^	K_0_, m^2^	δ_cr_
0.15	127.5	106.7	0.84
0.25	71.1	57.5	0.81
0.40	27.6	19.8	0.72

F is the surface density; N and K_0_ are the coefficients of the Equation (9) and are needed for the calculation of the permeability coefficient of the materials with known porosity; δ_cr_ is the minimum value of the porosity coefficient, limiting the scope of the presented functional model.

## Data Availability

Data are contained within the article.
